# Provident and Improvident Hospitals

**Published:** 1888-07-21

**Authors:** 


### The Hospital

A Weekly Institutional Journal of

**Science, Medicine, Nursing, and Philanthropy.**

*For Hospitals, Asylums, Medical Practitioners, Students, Nurses, Families, Parishes, Congregations, and the Charitable Public.*

Vol. IV. No. 95.] [July 21, 1888.

### PROVIDENT AND IMPROVIDENT HOSPITALS.

The Prince and Princess of Wales formally opened the new buildings of the Great Northern Central Hospital on Tuesday last. The occasion was one of great moment to the poorer inhabitants of North London, and has been long looked forward to with great interest by all classes. The district in which the new hospital is built has been singularly destitute of such accommodation hitherto; and it is mainly owing to the public spirit and unwearied devotion of some of its leading inhabitants that the carrying out of so great a work as the erection of a large general hospital has been rendered possible. The Prince and Princess of Wales have wisely recognised the public services thus rendered; and their prompt response to the loyal request of the representatives of the parish of Islington that they should be present and open the new buildings has given universal satisfaction.

The new hospital is making a forward movement on right lines. It aims at meeting the wants, not of a class merely, but of all whose circumstances render a temporary use of hospital treatment desirable and necessary. Whilst it wishes to avoid pauperising patients by a free system which acknowledges no checks of any kind, it is equally resolved not to interfere with the legitimate interests of private practitioners. It has certain wards for paying patients; but these we believe it is not intended to use in competition with the medical men of the district, or by way of adding to the financial resources of the institution. There are, in a large district like that of Northern London, thousands of young men and women, clerks, shop assistants, and others of the same class. The houses in which they reside are often occupied by overworked and poorly-paid landladies, and still more overworked and under-paid domestic helps. The addition of a patient and a sick-room to the already almost insupportable burden of the work of the house is frequently the last straw which breaks the camel's back. Under such circumstances the removal of the patient to a hospital, where he may pay all or a portion of the expenses incurred for his treatment, is hailed as a relief by all parties; by the landlady who cannot possibly add the responsibilities of a sick nurse to her other duties, and still more by the patient who is removed from bustle to quiet, and from the partial and fitful attention of incompetent and often unwilling hands to the care of those whose one business and pleasure it is to minister to his necessities and to promote his restoration to health. The only person who might object is the doctor, whose paying patient is thus removed from his care. But the hospital anticipates this contingency also, and provides that every patient occupying its paying wards shall, if he so chooses, be treated by his private medical attendant.

With regard to the out-patient department, this hospital has a system of its own. The system is one in which careful inquiry is combined with small payments. The established rule is to obtain full knowledge of every person, old or young, who makes use of the department. In those instances where it is found that the applicant is able to pay the moderate fee of the ordinary practitioner, treatment is absolutely refused. In the cases where it is ascertained that an ordinary doctor's fee is impossible, and these, we are assured, constitute the overwhelming majority, treatment is given. But most of these really poor patients it is found are able to contribute a very small sum—a few pence usually—and this small payment is cheerfully submitted to, and is found almost sufficient to cover the cost of the drugs used by the hospital. The aim of the Great Northern Central is to be on the one hand a free hospital for the relief of the really poor, and, on the other, to absolutely prevent the abuse of charity by the non-necessitous. The practice adopted is a kind of compromise between the paying and the non-paying systems, and is the result of repeated and candid discussions by the highly intelligent and conscientious committee of the institution, part of whom favour the pay whilst the others incline to the free system.

At the distance of about two miles from the Great Northern Central, in a direction due east, is the Metropolitan Hospital, formerly the Metropolitan Free. This also is a new building. Here a departure of a different and entirely novel character has been made. The hospital frankly declares itself a provident institution. The necessitous poor are publicly invited to become members when in health precisely as they might be asked to become members of a friendly society or of a coal or blanket club. Weekly payments are taken all the year round; definite hours are fixed when the medical officers of the provident department may be consulted at the hospital; a system of home visitation by thoroughly experienced practitioners is in full operation. The whole district supplied by the hospital is definitely mapped out, and there is an evident and resolute attempt to provide for the necessitous poor prompt and thoroughly efficient medical relief. Here again, however, there is a fixed determination to prevent the abuse of charitable resources by the non-necessitous. A wage limit is fixed, which of course is different for single and married men. Those whose wages exceed the limit prescribed are not eligible, except in unusual circumstances, for the provident department. All the provident members are not merely entitled to treatment as out-patients and at their own homes, but in case of serious illness have priority over all other applicants as in-patients at the hospital. The system is said to work wonderfully well. The poor of the district have availed themselves of it with great eagerness; for although it has only been in operation a few months, there are no fewer than 6,000 members at the present time, and the number is steadily increasing.

As a set-off to the satisfaction we feel at the initiation of the hospital provident system under such favourable auspices, there is to be mentioned the undoubted fact that a considerable number of medical practitioners, whose work lies within the district supplied by the hospital, express warm disapprobation of the movement. Perhaps they are at present more frightened than hurt. It is hardly likely that in so short a time any appreciable effect can have been produced upon their professional earnings. We would ask them to wait at least a year or two before they pronounce decidedly one way or the other. If, in the long run, it should be proved beyond doubt that a serious diminution of income results to neighbouring medical men from the action of the hospital, the whole question will have to be reconsidered from their point of view. We cannot see it to be desirable that public charity given for the relief of one class should be so employed as to bring about the ruin of another.

The two hospitals, whose position and work we have so briefly and rapidly sketched, have this marked claim to public consideration, that each is placed in the very centre of a district where hospital accommodation is urgently needed. Each, moreover, is under the control of a committee which is resolved to adapt its operations to the necessities of the circumstances and of the times. Compared with certain large and small hospitals which might be named, which are crowded together at the West End, and which because they look to the wealthy and uncritical population of the West End for support, run riot in their management and defy public opinion, the Great Northern Central and the Metropolitan Hospital occupy a very striking elevation indeed. They supply the needy of their own districts with that which they imperatively need, they draw their resources largely from the poor and middle classes of their own districts, they are managed almost entirely by men residing in those districts, who are conversant with the necessities of their less fortunate fellows; and they furnish centres of public interest and activity of the most wholesome and beneficial kind. It will be necessary at some future time to deal resolutely with the hospitals which crowd the West End, and leave the poor of the outlying quarters to their fate. In the meantime, we commend to the thoughtful benevolence of the philanthropic these two institutions, which on so many grounds deserve the confidence and support of the public.

